# Integrating transcriptome and proteome profiles to compare carcass and meat quality traits between Songliao and Songlei black pigs

**DOI:** 10.3389/fnut.2026.1710841

**Published:** 2026-01-29

**Authors:** Yunpeng Zhang, Qi Zhang, Suthar Teerath Kumar, Jing Xu, Yupeng Xie, Zhihao Wang, Wu-Sheng Sun, Li Pan, Yuan Zhao, Shu-Min Zhang

**Affiliations:** 1 Key Laboratory of Animal Production, Product Quality and Security, Ministry of Education, College of Animal Science and Technology, Jilin Agricultural University, Changchun, China; 2 Institute of Animal and Veterinary Sciences, Jilin Academy of Agricultural Sciences, Changchun, China; 3 Jilin Provincial Key Laboratory of Animal Microbiological Vaccine (Drugs) for Major Animal Diseases, Jilin Provincial Engineering Research Center of Animal Probiotics, Ministry of Education, Collage of Veterinary Medicine, Jilin Agricultural University, Changchun, China

**Keywords:** hybrid, intramuscular fat, proteomics, Songlei black pig, Songliao black pig, transcriptome

## Abstract

With the ongoing upgrade in consumption patterns, the pork market is shifting from a focus on quantity to an emphasis on quality, demanding higher intramuscular fat content alongside maintained growth rates. Crossbreeding between the Chinese lean-type Songliao black pig and the high-quality local breed Leixiang pig allows rapid integration of parental superior traits, resulting in hybrid vigor that effectively improves pork quality, growth performance, and economic benefits. We conducted transcriptomic and 4D microDIA proteomic sequencing analyses on the *longissimus dorsi* muscle tissue from hybrid offspring of purebred Songliao black pigs and Songliao × Leixiang black pigs. Extensive phenotypic analyses were performed on Songliao black pigs and Songlei black pigs using multiple trait indicators. Six pigs were selected and categorized into relatively high and low intramuscular fat groups. Integrated analysis of transcriptomic and proteomic data identified candidate genes within significantly annotated lipid-related pathways via KEGG, including *ACSL1*, *ACSL6*, *SREBF1*, *PLIN2*, *CEPT1*, *CPT1B*, *CPT1C*, and *ACSF3*. Among these, *CPT1B* was significantly associated with fatty acid metabolism pathways. By analyzing all significantly differential genes and proteins, six candidate genes were identified as key determinants of genetic variation in lipid deposition: *UCP3*, *CPT1B*, *LSMEM1*, *NEXN*, *PPP1R14C*, and *LOC100624149*. This preliminary exploratory multi-omics study provides a valuable resource for probing intramuscular fat deposition, aiming to support pork-trait improvement in breeding and to establish a fresh theoretical basis for clarifying the molecular mechanisms of meat-quality heterosis in Songlei black pigs.

## Introduction

With the improvement of living standards, the pork consumption market has shifted from quantity-driven to quality-oriented demand, placing greater emphasis on higher intramuscular fat (IMF) content. At the same time, there are stringent requirements regarding back fat and abdominal fat levels. Utilizing China’s lean-type pork breeds to crossbreed with high-quality local black pig varieties—known for their superior meat quality—has become a focal point in market development. Crossbreeding is an effective method to rapidly enhance livestock production performance by leveraging the dominant traits of parent breeds. It can generate hybrid vigor, improve production efficiency, and increase economic returns. Songlei black pigs are a binary hybrid line produced by crossing Leixiang sows with Songliao boars. The Songliao Black Pig, the first new pig breed in northern China with independent intellectual property rights, was approved by the National Livestock and Poultry Genetic Resources Committee in December 2009. It serves as a high-quality, efficient lean black pig parent breed. Leixiang pigs are known for their abundant intramuscular fat and tender meat, though they are relatively small in size and have a slow growth rate. The Songlei hybrid commercial pig not only yields high-quality black pork but also meets efficiency demands such as rapid growth and high lean meat percentage (1). Furthermore, it ensures a uniform black coat color in commercial herds, enhancing product consistency (1).

Transcriptomics is a comprehensive field focused on studying gene expression and regulation to uncover complex biological pathways and molecular mechanisms within regulatory networks. The most widely used method in transcriptomic research is transcriptome sequencing (RNA-Seq), which leverages next-generation high-throughput sequencing technologies. In studies related to pork quality, transcriptome sequencing is primarily applied to investigate differences in physiological status or production performance of skeletal muscle, as well as to compare meat quality traits at various developmental stages (2). Proteomics, building on transcriptomic data, examines gene expression differences at the protein level. It aims to analyze variations in protein expression, post-translational modifications, and protein-protein interactions that occur during cellular metabolism and other biological processes from a holistic perspective. Currently, proteomic technologies are being used to extensively explore the expression of differential proteins across various metabolic pathways, thereby facilitating the analysis of meat quality variations at the protein level (3). Through full-length transcriptome sequencing and bioinformatics analysis of skeletal muscle differences in Jiangquan black pigs, genes such as *TNNI2*, *TMOD4*, and *MYH1* were identified as potential key regulators affecting muscle development (4). In a comparison of quaternary hybrid lines—Jiaxing black pig (JXB) and Duroc × Duroc × Berkshire × Jiaxing black pig (DDBJ)—DDBJ exhibited superior growth and carcass traits, whereas JXB had significantly higher levels of flavor compounds, amino acids, and saturated fatty acids. Transcriptome analysis further identified differentially expressed genes, elucidating regulatory mechanisms underlying muscle development and meat quality, thereby providing a theoretical basis for genetic improvement in pig breeding (5). Proteomic analysis of the *longissimus dorsi* (LD) muscle in Shaziling and Large White pigs revealed 23 differentially expressed proteins, including triose phosphate isomerase (TPI) and enolase 3 (ENO3), which may be involved in fatty acid metabolism, glycolytic pathways, and skeletal muscle growth (6). Given that meat quality is influenced by multiple factors, single-omics approaches often fail to fully elucidate the underlying mechanisms. Consequently, multi-omics integration has become a predominant strategy in recent research. For instance, a combined transcriptomic and proteomic study comparing muscle tissues among Tibetan, Landrace, and Large White pigs identified 209 synergistically differentially expressed genes at both mRNA and protein levels in Tibetan pigs. These genes were primarily enriched in amino acid metabolism and energy pathways. Among them, 20 genes involved in muscle fiber formation were found to potentially significantly influence postnatal growth rate and body weight (7). Similarly, transcriptome and proteome analyses of the LD muscle in Nanyang black pigs with varying intramuscular fat content uncovered 25 candidate genes associated with genetic variations in lipid deposition, offering new insights into the mechanisms of fat accumulation (8). As nutrigenomics reveals its potential to transform global health, the link between genotype and phenotype is gaining increased attention. Genome-wide association studies (GWAS) have identified multiple genes linked to polygenic obesity, most of which participate in the leptin–melanocortin pathway regulating food intake (9). Moreover, the severity of obesity and alterations in fat metabolism are significantly influenced by other individual genetic variants, including those associated with obesity (e.g., *FTO* and *NPY*) and type 2 diabetes (e.g., *TCF7L2* and *IRS1*) (10).

The rapid advancement of omics technologies and their expanding application in pork quality research have greatly enriched multidimensional datasets related to fat deposition. This study systematically compares the expression of lipid metabolism-related genes and proteins in Songliao black pigs and their hybrid offspring, Songlei black pigs, using integrated transcriptomics and proteomics. We further examine how hybridization-induced differentially expressed genes participate in and influence nutritional metabolic pathways—such as fatty acid metabolism—to elucidate the molecular mechanisms by which hybridization affects meat quality from a gene-nutrient interaction perspective, and to uncover potential regulatory networks underlying fat metabolism.

## Materials and methods

### Ethical statement

A total of 112 experimental animals consisting of Songliao black pigs and Songlei black pigs were provided by Jilin Pegasus Animal Husbandry Co., Ltd. All slaughtering and sampling procedures were conducted under strict supervision and complied with relevant ethical standards. The animal care protocol was approved by the Animal Welfare Committee of Jilin Agricultural University (Approval No.: SYXK-2023-06-09-001).

### Sample collection

This study included 112 eight-month-old fattened pigs: Songliao black pigs (control group) and first-generation Songlei hybrid pigs (experimental group) (Supplementary Table 1). The nutrition value of diet were in accordance with the national standard (NY/T 65-2021) (Supplementary Table 2), and the pigs were provided with *ad libitum* access of fed and water under the same conditions for 45 days. The ambient temperature was maintained at 20–25°C with relative humidity of 60–65%. All pigs were euthanized by electrical stunning at 240 days of age after being fasted overnight. The effectiveness of stunning was confirmed by the immediate absence of the corneal reflex, pupillary dilation unresponsive to light, and the cessation of rhythmic breathing. Subsequently, exsanguination was performed as a secondary physical method to ensure death, in accordance with the American Veterinary Medical Association (AVMA) Guidelines for the Euthanasia of Animals. Within 45 min post-slaughter, a 500 g sample of the LD muscle was collected extending posteriorly from the anterior aspect of the third-to-last thoracic vertebra and stored at 4°C for meat quality assessment. Additionally, a separate 20 g sample of the LD was rapidly frozen in liquid nitrogen and maintained at –80°C for subsequent molecular analyses.

### Determination of carcass and meat traits

Carcass and meat quality traits were determined in accordance with the Chinese national Operating Procedures for Livestock and Poultry Slaughtering (GB/T 17236–2019) and the Technical Regulations for Pork Quality Determination (NY/T 821–2019). The specific measurements and methods were as follows: Live weight: Body weight after a 24-h fast prior to slaughter. Carcass weight: Body weight after slaughter, bleeding, evisceration, and removal of hair, head, hooves, tail, kidneys, and leaf fat. Carcass length: Distance from the anterior edge of the pubic symphysis to the anterior edge of the first cervical vertebra. Backfat thickness: Measured perpendicularly at the mid-point of the 6th–7th ribs using a vernier caliper. Meat color: Evaluated on the cross-section of the LD muscle within 24 h post-slaughter. Marbling score: Assessed based on the distribution of intramuscular fat within the cross-section of the LD muscle. IMF content: Approximately 100 g of fresh LD muscle was dissected, homogenized, and dried for Soxhlet extraction. Shear force: Meat samples were cooked to an internal temperature of 70°C, cooled, and subsequently analyzed on a texture analyzer (TA.Gel) with cylindrical cores taken parallel to the muscle fibers. Individuals were ranked by IMF content, with the top and bottom 10% assigned to high- and low-meat-quality groups, respectively. Extreme phenotypes from these tails (high-IMF, *n* = 3; low-IMF, *n* = 3) were then selected for multi-omics sequencing.

### Transcriptome sequencing analysis

Total RNA was isolated from LD muscle tissue using the TRIzol method. For each experimental and control group, six individual samples were collected, with each group comprising three biological replicates. High-quality RNA samples, defined by an RNA integrity number (RIN) > 7.0, were selected for cDNA library construction. Sequencing was conducted on an Illumina platform in paired-end 150 bp (PE150) mode. To minimize potential batch effects, library preparation and sequencing of all samples were performed in a fully randomized order. Raw sequencing data were quality-controlled with FastQC (v0.11.9), and low-quality sequences were filtered out using Trimmomatic (v0.39). The resulting high-quality reads were aligned to the porcine reference genome (Sscrofa11.1) using HISAT2, and gene expression levels were quantified in FPKM units with featureCounts. Differential gene expression analysis was performed using DESeq2, in which raw read counts were normalized via the built-in median-of-ratios method to account for differences in library size and RNA composition. Significantly differentially expressed genes were identified using thresholds of |log_2_ fold change (FC)| > 1 and an adjusted *P*-value (*P*) < 0.05 (2).

### 4 D microDIA proteomics analysis

Total protein was extracted from the six individual LD muscles using SISPROT lysis buffer, and the concentration was determined with a BCA assay. A total of 10 μg of protein was subjected to enzymatic digestion and desalting using a C18 column. The peptides were separated on a nanoElute UHPLC system equipped with a C18 reverse-phase column, using a gradient elution with 0.1% formic acid in water and acetonitrile. Mass spectrometry data were acquired in ddaPASEF mode on a timsTOF Pro 2 instrument, with an m/z range of 100–1,700 and an ion mobility range of 1/K_0_0.7–1.4 Vs/cm^2^. DIA window set to 32 variable windows in ion mobility-mass-charge ratio two-dimensional space, collision energy 27–45 eV (linearly adjusted with mobility), accumulation time 100 ms. Raw data were processed using DIA-NN (v1.8.1). The spectral library was generated using a prediction strategy (uniprot_proteomeUP000008227_pig.fasta; trypsin cleavage, up to 2 missed cleavages; fixed modifications: Cys carboxymethylation; variable modifications: Met oxidation, N-terminal acetylation). Quantification based on MaxLFQ algorithm (requires ≥ 2 unique peptides), and a false discovery rate (FDR) ≤ 1%. Quantitative data were analyzed in Perseus (v2.0.7) following log_2_-transformation and subsequent Student’s *t*-tests. Owing to the exploratory nature and limited sample size of this study, differential proteins were screened using a significance threshold of FC ≥ 1.5 or FC ≤ 0.667 together with a *P* < 0.05 (11).

### Functional annotation and enrichment analysis

Systematic biological approaches were applied to perform functional annotation and pathway enrichment analysis on multi-omics data, including differentially expressed genes (DEGs) and differentially expressed proteins (DEPs). Functional characterization was conducted based on the Gene Ontology (GO) database across three categories: biological process (BP), molecular function (MF), and cellular component (CC). Further pathway enrichment analysis was performed using the Kyoto Encyclopedia of Genes and Genomes (KEGG) database to identify significantly enriched metabolic and signal transduction pathways *(P* < 0.05) (2, 7).

### Protein–protein interaction network analysis

Protein–protein interaction (PPI) networks were constructed using the STRING database and subsequently visualized and analyzed in Cytoscape (6). Transcription factor (TF)–target gene networks were predicted using the NetworkAnalyst tool (12). We performed network visualization and all subsequent analyses in R using the “MASS” package, with comprehensive support from CNSknowall^1^ —an online platform specialized for integrated biomedical data analysis and visualization.

### Statistical analysis

Statistical analysis of carcass and meat quality traits were performed using GraphPad Prism 9.0. Data are presented as mean ± standard deviation (mean ± SD). Differences between groups were assessed using a two-tailed Student’s *t*-test. A *P* < 0.05 was considered statistically significant.

## Results

### Phenotypic analysis of different traits

**FIGURE 1:**
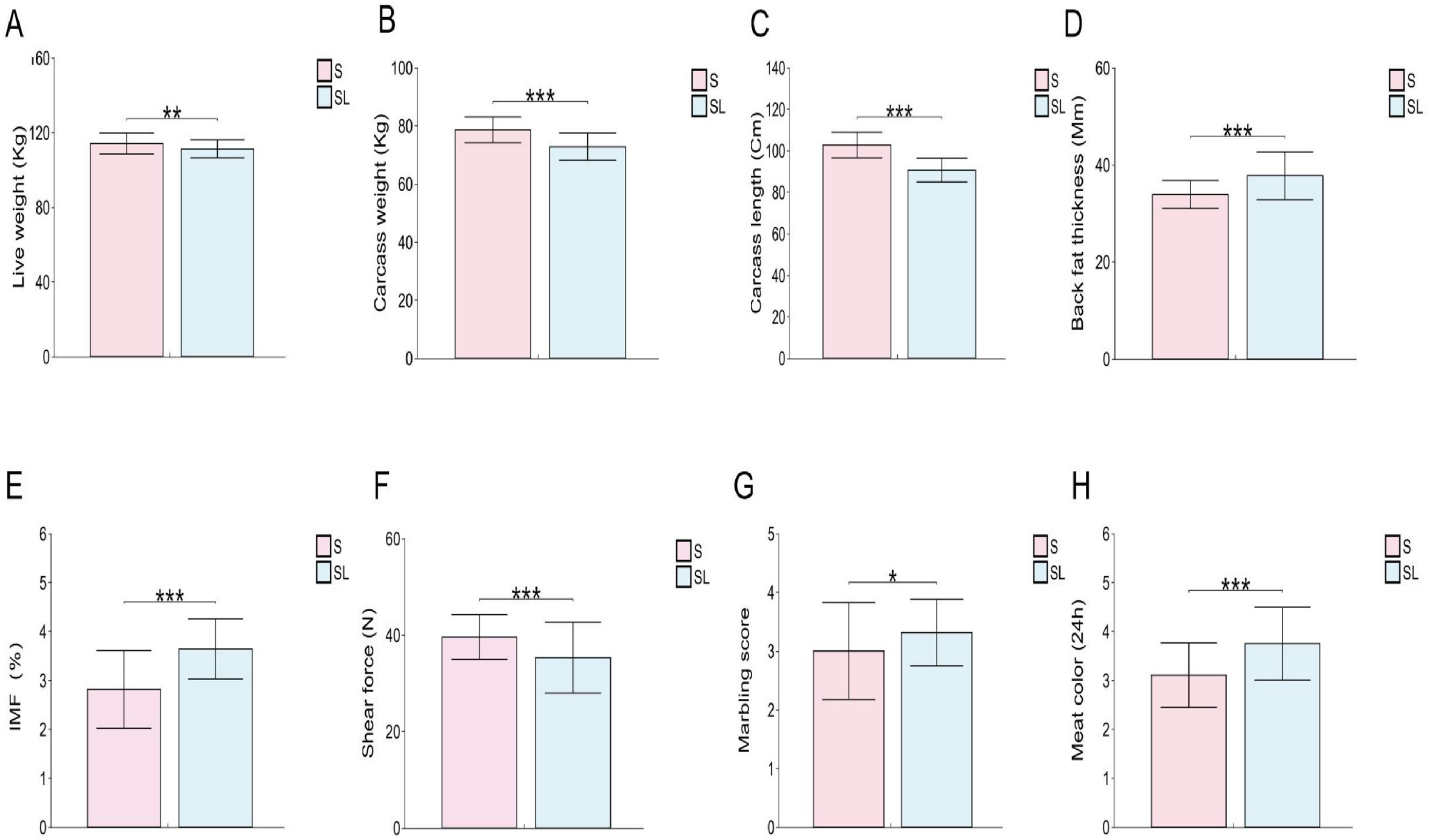
Carcass and meat quality trait analysis in Songliao black pig (S, red) and Songlei black pig (SL, blue) (*n* = 112). **(A)** Live weight. **(B)** Carcass weight. **(C)** Carcass length. **(D)** Back fat thickness. **(E)** IMF. **(F)** Shear force. **(G)** Meat color. **(H)** Marbling score. *P < 0.05; **P < 0.01; ***P < 0.001; *^ns^P* > 0.05.

Analysis of 112 individuals for carcass and meat quality traits revealed that the hybrid Songlei black pigs exhibited significantly greater values in meat color, marbling score, backfat thickness, and IMF content compared to the purebred Songliao black pigs (*P* < 0.05). In contrast, Live weight, carcass weight, carcass length, and shear force of the LD muscle were significantly lower in the hybrid group (*P* < 0.05) (Figure 1).

### Quality control of transcriptomic and proteomic data

**FIGURE 2:**
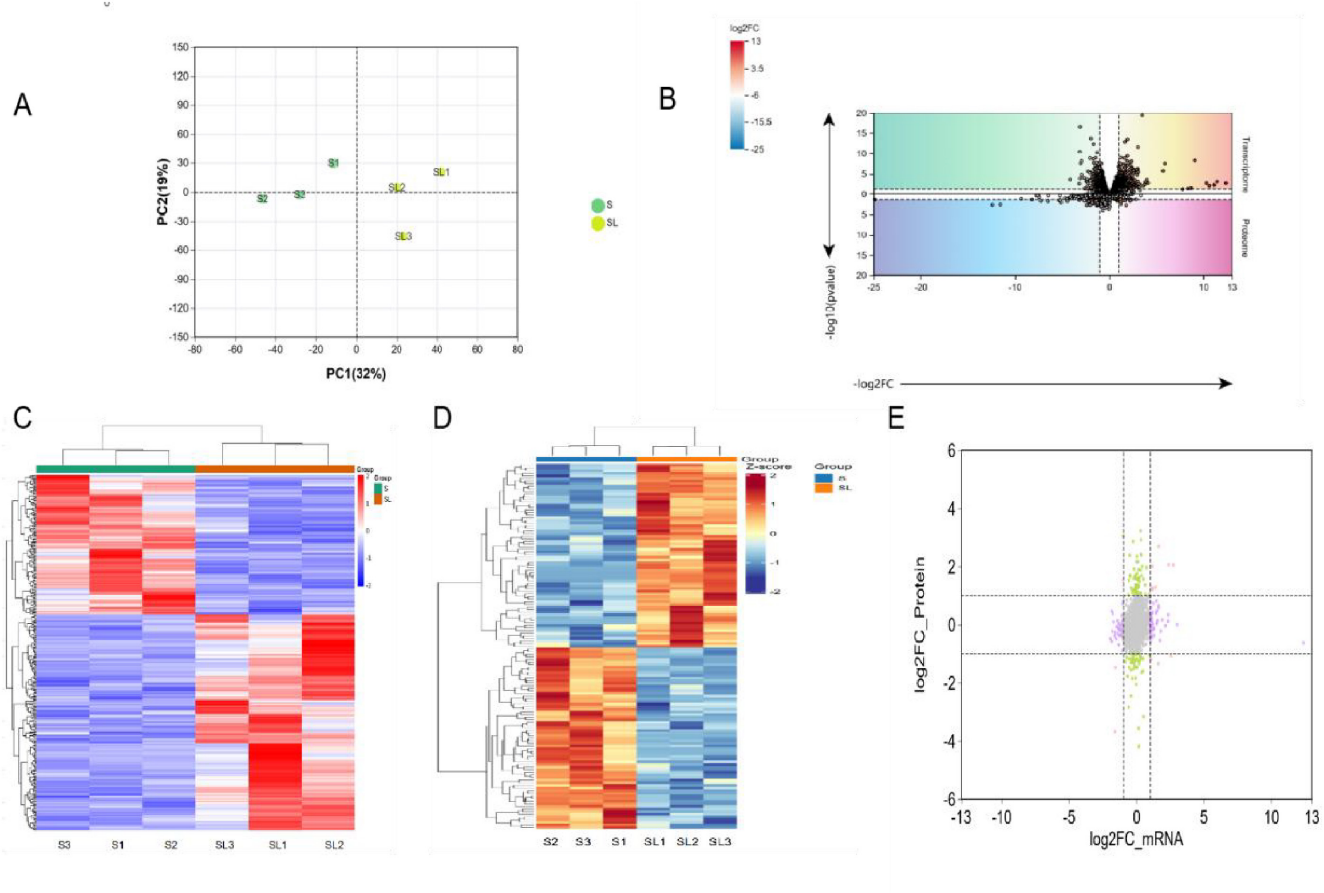
Transcriptomic and proteomic data analysis. **(A)** Principal component analysis (PCA) of gene expression profiles. **(B)** Volcano plots showing DEGs (upper panel) and DEPs (lower panel), colored by Log_2_FC (red for higher values, blue for lower values). **(C)** Heatmap of differentially expressed genes. **(D)** Heatmap of differentially expressed proteins. **(E)** Relationship between mRNA and protein expression. Quadrants I and III contain gene expression changes that are concordant between the transcript and protein levels, whereas Quadrants II and IV represent discordant changes.

Transcriptome sequencing yielded a total of 344.89 million high-quality reads, with an overall alignment rate of 99.97%. All samples exhibited excellent data quality, with clean reads ratios ranging from 97.79 to 98.48%. The Q20 and Q30 base percentages exceeded 98.80 and 94.34% (Supplementary Table 3). Based on FPKM quantification, 13,061 genes were stably expressed across all samples. Quality control analysis showed that the high intramuscular adipose group of Songlei black pigs (SL1, SL2, SL3) and Songliao black pigs (S1, S2, S3) showed significant grouping characteristics on the gene expression profile: whether based on FPKM value or gene expression, the samples of both groups were completely differentiated (Figure 2A). For proteomic analysis, DIA mass spectrometry was employed for in-depth profiling of muscle tissue proteomes. A total of 29,188 unique peptides corresponding to 3,289 proteins were identified across all samples. Quality assessment indicated that peptide length distribution was appropriate, with the majority of peptides falling within the 7–20 amino acid range, meeting standard quality control criteria. Enzymatic digestion efficiency was high, with 78.75% of peptides derived from single-enzyme cleavage sites, demonstrating the reliability of sample pretreatment.

### Differential gene and protein expression analysis

Using stringent criteria for differential expression, a total of 324 DEGs were identified. Among these, 197 genes were upregulated and 127 were downregulated in the SL group compared to the S group (Supplementary Table 4). Due to the absence of significant differentially expressed proteins after FDR correction (FDR < 0.05), we retained the exploratory findings generated under the less stringent cutoff of a raw *P* < 0.05. Based on this exploratory criterion, 139 DEPs were identified between the SL and S groups, with 70 proteins upregulated and 69 downregulated in the SL group relative to the S group (Figure 2B; Supplementary Table 5). DEGs and DEPss were analyzed using two complementary approaches: cluster analysis visualized in a heat map (Figures 2C,D) and a nine-quadrant diagram depicting expression changes between corresponding molecules (Figure 2E). The nine-quadrant diagram intuitively reveals the prevalent positive and negative regulatory relationships between proteins and their corresponding genes (13).

### GO enrichment analysis of DEGs and DEPs

**FIGURE 3:**
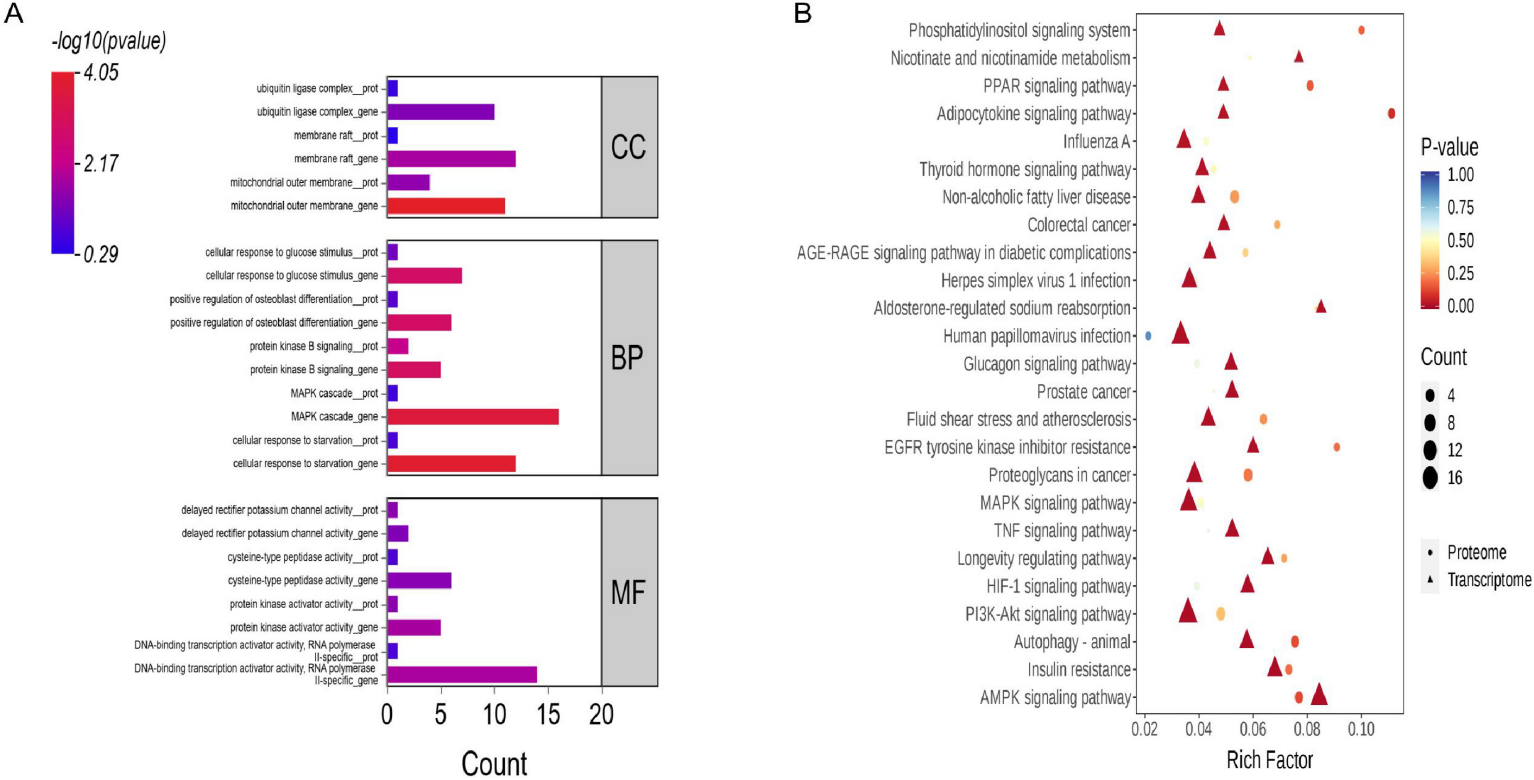
Functional analysis of DEGs and DEPs. **(A)** GO enrichment analysis. **(B)** KEGG enrichment analysis.

Based on GO enrichment analysis of DEGs and DEPs from transcriptomic and proteomic comparisons, commonly enriched functional terms shared between the two omics levels were identified and ranked in ascending order of enrichment significance (*P*). Commonly significantly enriched terms are displayed for each major GO category—Biological Process (BP), Cellular Component (CC), and Molecular Function (MF) (Figure 3A; Supplementary Table 6). The results indicate that both DEGs and DEPs are predominantly associated with the following three functional modules: Biological Process: significantly co-enriched in terms such as protein kinase B signaling, smooth muscle cell differentiation, and long-chain fatty acid transport; Cellular Component: primarily localized to structures including the mitochondrial outer membrane, voltage-gated potassium channel complex, and intercalated disc; Molecular Function: markedly enriched in activities related to transcriptional regulation and signal transduction, including DNA-binding transcription activator activity, protein kinase activator activity, delayed rectifier potassium channel activity, and cysteine-type peptidase activity.

### KEGG enrichment analysis of DEGs and DEPs

Based on KEGG enrichment analysis of DEGs and DEPs, 173 pathways were found to be co-enriched in both omics layers. A bar chart was generated to visualize the number of DEGs and DEPs enriched in each of these common pathways (Figure 3B). For KEGG categories containing more than 25 pathways, only the top 25 ranked by *P* are displayed, with transcriptome-based enrichment taking precedence in the selection. Among the results, 49 pathways were significantly enriched. These were primarily associated with: Lipid metabolism, including fatty acid metabolism, PPAR signaling pathway, AMPK signaling pathway, adipocytokine signaling pathway, and the PI3K-Akt signaling pathway; Tissue structure and homeostasis, such as autophagy and apoptosis; Oxygen-related metabolic processes, including the hypoxia-inducible factor-1 (HIF-1) signaling pathway and the cGMP-PKG signaling pathway. Notably, DEPs were significantly involved in 9 pathways, with major enrichment in ether lipid metabolism, fatty acid metabolism, selenocompound metabolism, and mitophagy—animal. Of these, fatty acid metabolism was a commonly significantly enriched pathway shared between both Transcriptome and proteome.

### Candidate gene analysis of lipid deposition-related pathways

**FIGURE 4:**
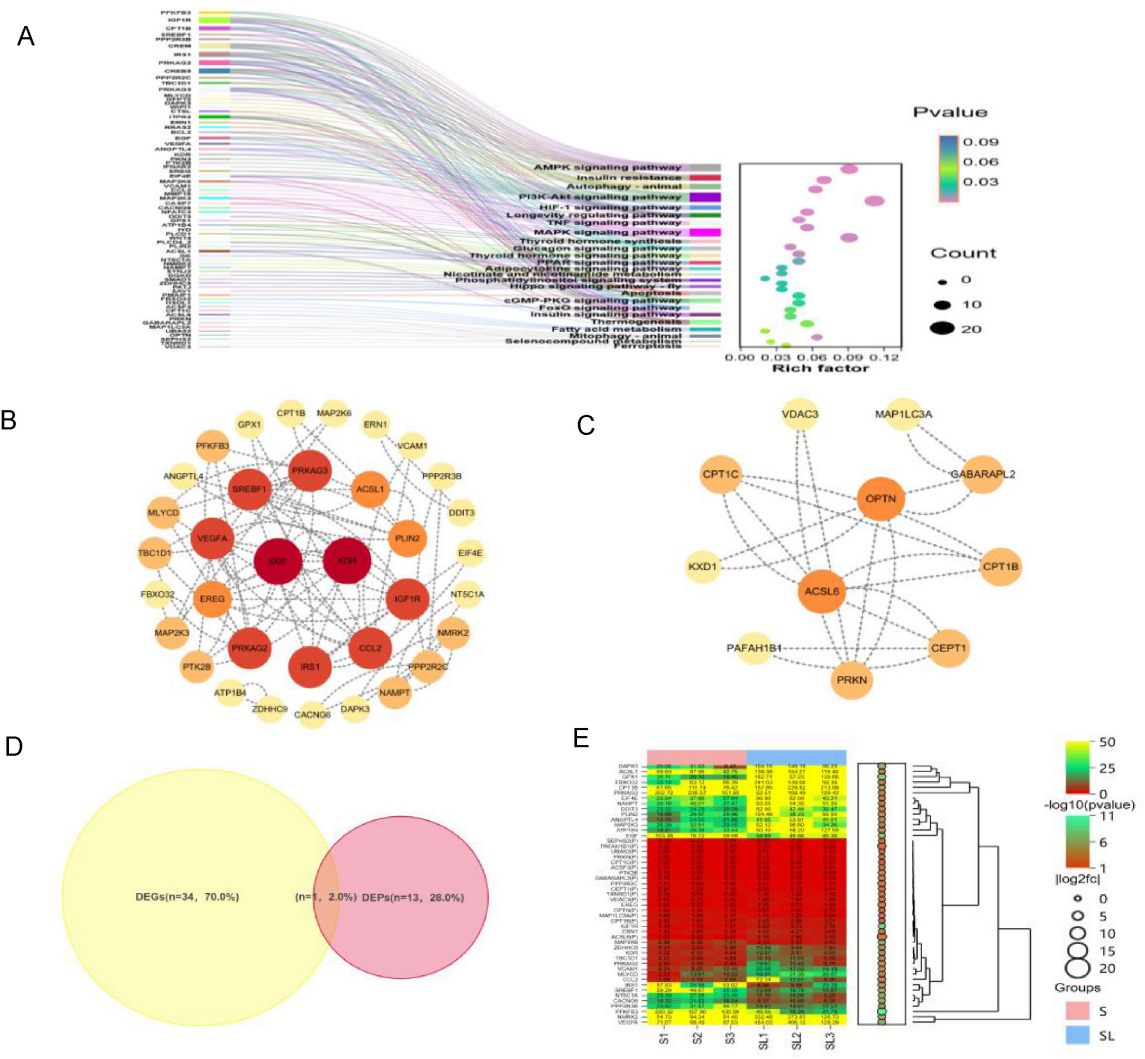
Candidate gene analysis of lipid deposition-related pathways. **(A)** KEGG enrichment analysis. **(B)** PPI network for Cytoscape visualizing DEGs. **(C)** PPI network for Cytoscape to visualize DEPs. **(D)** Overlapping of differentially expressed genes identified by transcriptome (yellow) and proteome (red). A total of 48 genes/proteins related to lipid metabolism were identified within significantly enriched pathways. CPT1B was differentially expressed at both the transcriptomic and proteomic levels and associated with lipid metabolism. **(E)** Expression levels of lipid metabolism-related genes across samples. The first bar on the right represents expression level, the second bar represents -log10 (*P*-value), and bubbles indicate the gene’s |log_2_FC|.

To further exploit the information from DEGs and DEPs, their functional and pathway characteristics were investigated through KEGG analyze. Based on the number of clustered genes and statistical significance (*P* < 0.05), a total of 48 candidate genes and proteins potentially involved in lipid deposition-related pathways were identified via KEGG enrichment (Figure 4A; Supplementary Table 7). Integration of these candidate genes from lipid-related pathways using the STRING database and Cytoscape network analysis revealed several functional categories (Figures 4B,C): Growth Factors, Cytokines, and Receptors: Epidermal Growth Factor (*EGF*), Vascular Endothelial Growth Factor A (*VEGFA*), Kinase Insert Domain Receptor (*KDR*), Insulin-like Growth Factor 1 Receptor (*IGF1R*), and C-C Motif Chemokine Ligand 2 (*CCL2*); Lipid Metabolism and Lipid Droplets: Acyl-CoA Synthetase Long Chain Family Member 1/6 (*ACSL1*/*6*), Sterol Regulatory Element Binding Transcription Factor 1 (*SREBF1*) and Choline/Ethanolamine Phosphotransferase 1 (*CEPT1*); Fatty Acid Oxidation and Transport: Carnitine Palmitoyltransferase 1B/C (*CPT1B/C*); Energy Sensing and Signaling: Protein Kinase AMP-Activated Non-Catalytic Subunit Gamma 2/3 (*PRKAG2/3*) and Insulin Receptor Substrate 1 (IRS1); Autophagy and Mitophagy: Parkin RBR E3 Ubiquitin Protein Ligase (*PRKN*), Optineurin (*OPTN*) and GABA Type A Receptor Associated Protein Like 2 (*GABARAPL2*) (Supplementary Table 8). Notably, the *CPT1B* gene was annotated to the fatty acid metabolism pathway in both the transcriptome and proteome analyses and was highly expressed in the high intramuscular fat group (Figures 4D,E).

### Analysis of common DEGs and prediction of transcription factors

**FIGURE 5:**
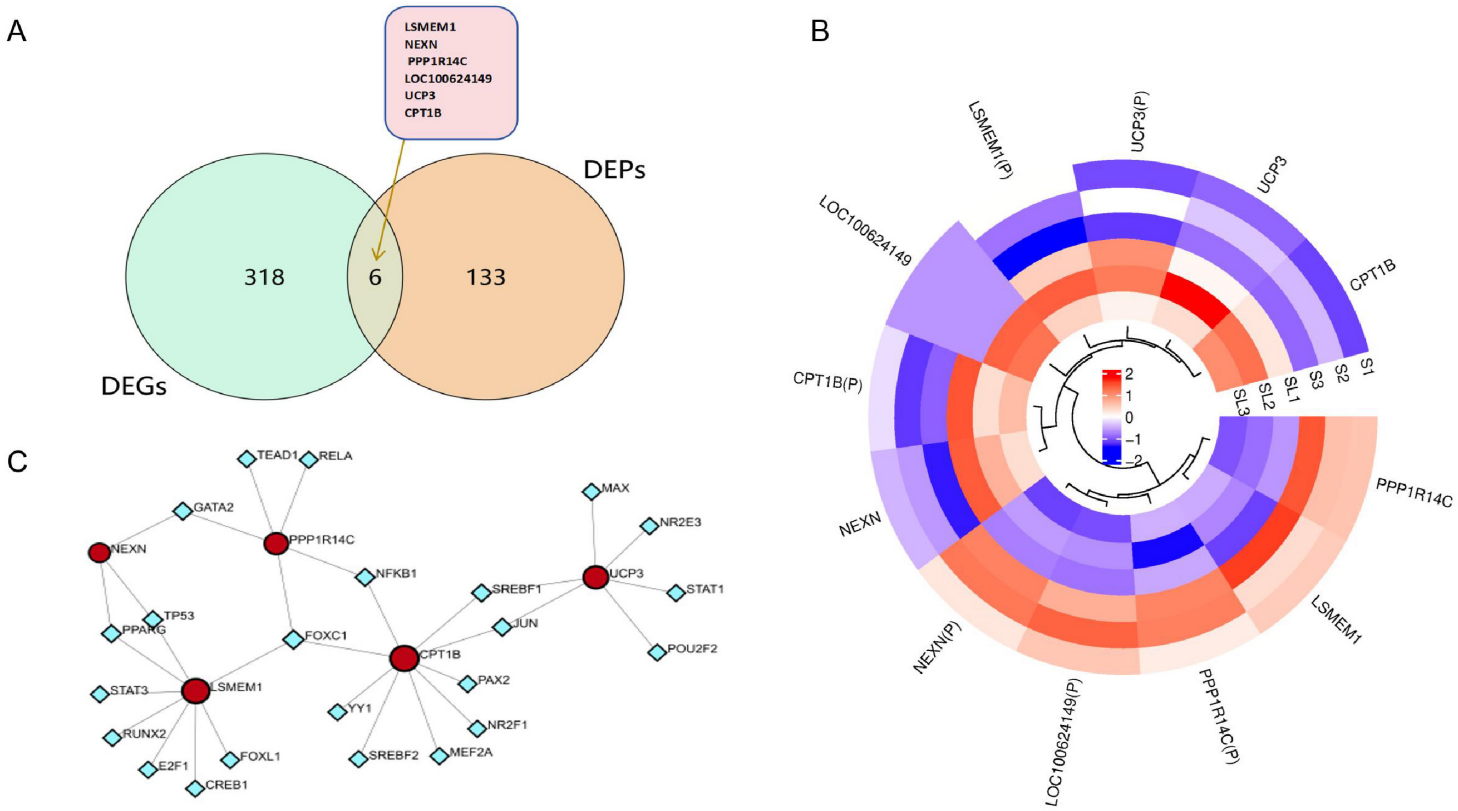
Functional analysis of differentially expressed genes. **(A)** Venn diagram DEGs (green) and DEPs (yellow). **(B)** Clustering heatmap of common differentially expressed genes, with differentially expressed proteins labeled with the suffix “(P).” **(C)** Prediction of upstream transcription factors (blue).

Transcriptomic mRNA data were integrated with proteomic protein identification results to establish correspondence between mRNA and protein levels. Subsequently, all differentially expressed genes and proteins were subjected to Venn analysis. This analysis revealed the number of common and unique proteins or genes across different comparison groups, identifying six common genes: *LSMEM1*, *NEXN*, *PPP1R14C*, *LOC100624149*, *UCP3* and *CPT1B* (Figures 5A,B). Furthermore, upstream regulatory transcription factors of these common differentially expressed genes were predicted using the NetworkAnalyst online tool (Figure 5C).

### Functional analysis of common DEGs

Based on an in-depth analysis of functional annotations from GO and KEGG for six co-expressed differentially expressed genes, only entries that are statistically significant (e.g., *P* < 0.05) or possess relevant biological significance are reported. The *p*-value is derived from the enrichment analysis results of the gene set. GO analysis showed that *NEXN*, *LOC100624149*, *UCP3* and *CPT1B* were associated with specific biological processes. In contrast, *LSMEM1* and *PPP1R14C* were not annotated to any specific pathways or processes. GO analysis indicated that *LOC100624149* was annotated to the translation process (GO:0043043); *NEXN* was involved in muscle tissue development (GO:0060537) and striated muscle tissue development (GO:0014706) (14); *UCP3* was associated with the cellular response to decreased oxygen levels (GO:0036293), response to superoxide (GO:0000303), and response to steroid hormones (GO:0048545); Both *UCP3* and *CPT1B* were co-annotated to the fatty acid metabolic process (GO:0006631) (15), mitochondrial transport (GO:0006839), and mitochondrial transmembrane transport (GO:1990542) (16) (Supplementary Table 9). KEGG analysis further demonstrated that *CPT1B* was annotated to multiple lipid metabolism pathways, including the AMPK signaling pathway, insulin resistance, glucagon signaling pathway, PPAR signaling pathway, adipocytokine signaling pathway, thermogenesis, fatty acid metabolism, and fatty acid degradation (17); *LOC100624149* was annotated to the nuclear transport pathway (ko03013) (Supplementary Table 10).

## Discussion

Amidst evolving consumption patterns and structural adjustments within the swine industry, high-quality black pigs have gained significant popularity due to their distinctive meat flavor and health benefits. The Songlei Black Pig represents an innovative hybrid breed developed by Leixiang sow and Songliao Black Pig as parental lines. To achieve simultaneous improvements in both growth performance and meat quality, this study evaluated carcass characteristics and meat quality traits in both the purebred Songliao Black Pig and the hybrid Songlei Black Pig. Results indicated that, at the same age, the Songlei Black Pig exhibited superior meat color, marbling, intramuscular fat content, and tenderness compared to the Songliao Black Pig, although its growth performance and backfat thickness were slightly inferior. Subsequent integrated transcriptomic and proteomic analyses identified key regulatory genes and pathways associated with growth and meat quality traits. Six common differentially expressed genes were found to be significantly enriched in lipid metabolic pathways in both omics layers. Among these, the gene *LOC100624149*—previously uncharacterized—was annotated to translation-related processes (GO:0043043) and nuclear transport pathway (ko03013), suggesting a role in protein synthesis. We hypothesize that this gene may play a regulatory role during the initiation phase of translation, potentially forming a novel regulatory axis: translation initiation regulation-expression of lipid-metabolism-related proteins -lipid deposition. Thus, *LOC100624149* could serve as a molecular marker or a potential intervention target for precisely modulating intramuscular fat content through regulation of its expression or activity.

Fat deposition plays a decisive role in pork quality and serves as a core indicator for meat quality evaluation, with multifaceted impacts on various meat attributes. Uncoupling protein 3 (*UCP3*), a member of the mitochondrial inner membrane anion transporter family, regulates cellular thermogenesis and energy metabolism by inducing proton leakage and uncoupling oxidative phosphorylation, thereby promoting energy dissipation as heat (18). Cold stimulation upregulates *UCP3* expression via PPARγ activation in brown/beige adipocytes, promoting lipolysis and thermal adaptation (16). Studies have confirmed that cold stress activates the cGMP–PKG, PPAR, and AMPK signaling pathways through the gut–blood–fat axis, while enriching lipid degradation-related genes—including *CLPS*, *PNLIPRP1*, *CPT1A/B*, and UCP3-leading to upregulated *UCP3* expression, browning of white adipose tissue, and thermogenesis (19). In this study, Songliao black pigs, derived from Northeast Chinese indigenous breeds, and Leixiang sows, originating from Tibet with Tibetan pig ancestry, both exhibit strong cold adaptability. Studies have found that Tibetan pigs and folk pigs adapt to cold climate depend on browning of white fat and upregulated expression of *UCP3*, indicating that the *UCP3* gene has the ability to produce heat in pig fat (20). *UCP3* enhances fatty acid oxidation and mitochondrial function in skeletal muscle, reduces reactive oxygen species (ROS) production, promotes oxidative fiber formation, and consequently facilitates IMF deposition. Oxidative fibers are rich in myoglobin and phospholipids, further supporting IMF accumulation (21). Consistent with these mechanisms, *UCP3* in this study was annotated to responses to oxygen deficiency and superoxide. Its upregulation suggests activation of antioxidant defenses against ROS-induced oxidative stress. Furthermore, *UCP3* may act through a compensatory mechanism analogous to a fatty acid synthase inhibitor, improving metabolic efficiency and promoting fat re-accumulation (22). Genetic studies localize the porcine *UCP3* gene to the SSC9p21–p24 region. Polymorphisms in this gene—such as a 9-bp repeat mutation in the 3′UTR—are significantly associated with backfat thickness, IMF content, and meat quality traits such as temperature and juiciness (23). Carnitine palmitoyltransferase 1B (*CPT1B*) is a key rate-limiting enzyme in skeletal muscle and adipose tissue that regulates long-chain fatty acid β-oxidation. Its activity directly influences mitochondrial lipid oxidation capacity and cellular energy metabolism homeostasis (24). *CPT1B* facilitates the transport of fatty acids into the mitochondrial matrix for oxidative degradation by catalyzing the formation of acylcarnitine. This process is transcriptionally regulated by nuclear receptors such as PPARα/δ and responds to physiological signals including cold stress and nutritional status (25). Molecular mechanistic studies indicate that upregulation of *CPT1B* expression promotes lipid accumulation and triglyceride synthesis, enhances IMF deposition, and is positively correlated with the metabolic characteristics of oxidative muscle fibers (type I) (26, 27). Functional studies have demonstrated that loss of *CPT1B* leads to impaired mitochondrial oxidative function, reduced cold tolerance (28), whereas its overexpression significantly increases lipid accumulation (29). Multiple studies suggest that *CPT1B* plays a central role in metabolic disorders such as aging, obesity, and insulin resistance (30–32). Its expression level and activity represent a potential target for metabolic regulation, correlating with backfat thickness, fatty acid composition, and thermogenic capacity (33, 34). In this study, both *UCP3* and *CPT1B* were annotated to the GO terms fatty acid metabolic process (GO:0006631), mitochondrial transport (GO:0006839), and mitochondrial transmembrane transport (GO:1990542). CPT1B was also annotated to multiple lipid metabolism-related pathways, including the AMPK signaling pathway, insulin resistance, glucagon signaling pathway, PPAR signaling pathway, adipocytokine signaling pathway, thermogenesis, fatty acid metabolism, and fatty acid degradation. It is noteworthy that fatty acid metabolism pathways were consistently and significantly enriched in both transcriptomic and proteomic analyses. Specifically, expression levels of *UCP3* and *CPT1B* in the experimental group were approximately 2.31- and 2.36-fold higher, respectively, compared with the control group. Their mean expression values across individual Songlei black pig samples reached 622.19 and 200.55, respectively. These findings indicate that Songlei black pigs exhibit a distinct transcriptional and translational upregulation of key lipid-metabolism-related genes. The coordinated and pronounced elevation of both *UCP3* and *CPT1B* further suggests their potential synergistic role in modulating lipid metabolism and mitochondrial thermogenesis, which may collectively influence skeletal muscle energy metabolism and associated meat-quality traits.

The rapid growth and bone development in pigs are inevitable outcomes of the modern swine industry’s pursuit of high efficiency, yet their implications for production are multifaceted. While directly enhancing production efficiency, feed conversion rate, and economic returns—core indicators of industrial advancement—these traits also entail complex biological trade-offs. Leucine-rich single-pass membrane protein 1*(LSMEM1*), located at 7q31.1, encodes an approximately 34 kD leucine-rich single-pass membrane protein. This protein mediates protein-protein interactions via its leucine-rich repeat domain and is implicated in maintaining membrane integrity and facilitating intercellular signaling. It is highly expressed in brain tissue, cardiac muscle, and skeletal muscle, and has been associated with the differentiation processes of neural and muscular cells. Through multi-omics integration analysis, 54 key genes regulating skeletal muscle development were identified, among which *LSMEM1* and *STEAP4* were found to be significantly upregulated. Subsequent motif analysis suggested that *LSMEM1* may be regulated by the transcription factor MEF2C, offering novel insights into the molecular mechanisms underlying skeletal muscle development (35). Further studies indicate that environmental endocrine disruptors such as bisphenol A (BPA) and bisphenol S (BPS) can interfere with steroid synthesis in sheep granulosa cells (GC). Specifically, BPA upregulates *LSMEM1* and *JUN* expression, affecting the MAPK signaling pathway. *LSMEM1* interacts with the transcription factor SRF to promote c-FOS expression, forming an AP-1 complex with c-JUN that collectively regulates apoptosis, proliferation, and differentiation (36). These findings suggest that *LSMEM1* may serve as a critical regulator influencing cell fate decisions. In the proteomic data of this study, *LSMEM1* was significantly upregulated. Notably, the hybrid Songlei Black Pig showed significantly reduced body size-related traits compared to the purebred Songliao Black Pig. This phenotypic divergence suggests that modulation of *LSMEM1* expression could represent a promising breeding strategy for developing new lines of Songlei Black Pigs with optimized growth rates.

Tenderness is a central parameter in pork quality grading systems, reflecting the force required to fracture muscle fibers during chewing. Pork with high tenderness is easier to chew, exhibits a delicate texture, and significantly enhances eating pleasure. Moreover, tender muscles typically demonstrate superior water-holding capacity, reduced cooking loss, and a richer sensory profile. Nexilin (*NEXN*) is a crucial component of cellular focal adhesions, playing a key role in cell–matrix adhesion by binding F-actin and mediating the linkage between integrins and the intracellular actin cytoskeleton. This protein influences cell migration, proliferation, and differentiation by modulating the stability of adhesion structures and facilitating signal transduction. Dysregulation of *NEXN* function is closely associated with impaired cell motility and related pathologies (37, 38). As a core element of the junctional membrane complex (JMC), *NEXN* is essential for maintaining JMC structural integrity, Ca^2+^ homeostasis, and contractile function (39). It localizes within quantitative trait loci (QTL) associated with IMF content and has been linked to adipogenesis and obesity (40). CRISPR/Cas9-generated NEXN−/− zebrafish models exhibit cardiac contractile deficits and stress-induced skeletal muscle disorganization, though compensatory upregulation of sarcomeric transcripts may partially stabilize muscle architecture, indicating an adaptive response to *NEXN* loss (41). Protein phosphatase 1 regulatory subunit 14C (PPP1R14C, also known as KEPI), expressed in the brain, heart, and skeletal muscle (42), participates in glycogen metabolism as a key regulator of GSK3β and may influence feed efficiency through energy metabolic pathways (43, 44). Integrated multi-omics analyses combining ATAC-seq and RNA-seq data revealed downregulation of *PPP1R14C* in the *longissimus dorsi muscle*. Given its role in myosin phosphatase inhibition and contraction regulation, this gene is postulated to influence muscle fiber differentiation and meat quality by modulating myofiber development—including diameter and cross-sectional area (12, 45). Proteomic data from this study revealed that the expression levels of PPP1R14C and NEXN in the longissimus dorsi muscle were downregulated by approximately 1.86- and 6.63-fold, respectively, in the experimental group, suggesting their potential involvement in regulating pork tenderness. Proteomic studies further indicate that *PPP1R14C* expression is significantly correlated with color and textural attributes of cooked pork, positioning it as a potential biomarker for cooking quality (11). These findings provide novel insights into the metabolic and epigenetic mechanisms underlying skeletal muscle development in pigs.

This study demonstrates that *UCP3* and *CPT1B* coregulate lipid metabolism and thermogenesis, collectively influencing fat deposition and meat quality. *CPT1B*, a key long-chain fatty acid transporter, represents a potential target for n-3 PUFA-based nutritional interventions, while *UCP3* offers a mechanistic basis for modulating muscle energy partitioning via dietary strategies (46). In muscle development, *LSMEM1* likely regulates fiber formation through the transcription factor MEF2C, whereas *NEXN* and *PPP1R14C* contribute to structural integrity and tenderness, respectively. The newly identified gene *LOC100624149* may participate in lipogenic protein synthesis via translational initiation control, revealing a previously unexplored layer of lipid metabolic regulation. From a nutrigenomic perspective, these findings offer practical insights: *CPT1B* and *UCP3* expression profiles may guide precision nutrition strategies to improve meat traits; genetic variations in these genes could serve as markers for molecular breeding; and pathway-informed feed formulations may enhance both production efficiency and meat quality (47). Together, these results establish a gene–nutrition framework for optimizing meat production in livestock.

In this study, our multi-omics analysis revealed a limited overlap between genes exhibiting significant differences at the transcriptomic and proteomic levels. While this discrepancy presents a common challenge in integrative omics research, it simultaneously offers a critical insight into the underlying biological regulation. The observed decoupling between mRNA expression and protein abundance strongly indicates the presence of extensive post-transcriptional and translational regulatory mechanisms, which are pivotal in determining the ultimate phenotypic outcomes. Although the current sample size (*n* = 3 per group) is insufficient for robust population-level statistical inference, it represents a valid and established exploratory design for in-depth mechanistic investigation and hypothesis generation. Importantly, this discussion does not undermine but rather reinforces the core conclusion of our study: intramuscular fat deposition is a complex biological process subjected to multi-layered precision regulation across transcriptional, post-transcriptional, and translational stages. In future work, we plan to validate all key findings of this study in a larger, independent cohort, which we have designated as a priority for our subsequent research.

## Conclusion

In summary, this study employed a multi-omics comparative analysis of Songliao black pigs and their hybrid offspring, Songlei black pigs, to elucidate key molecular mechanisms underlying meat quality and growth traits. The results indicate that although Songlei black pigs exhibit slightly inferior growth performance in certain body measurement traits, they demonstrate superior meat quality, characterized by higher intramuscular fat content and improved tenderness. Through high-quality RNA-seq and 4D microDIA proteomic analyses, we identified 324 DEGs and 139 DEPs. Integrated transcriptomic and proteomic approaches further revealed six key candidate genes including *UCP3, CPT1B, LSMEM1, NEXN, PPP1R14C*, and *LOC100624149*, potentially associated with these traits. Subsequent GO and KEGG enrichment analyses indicated that the significantly differentially expressed genes and proteins were predominantly involved in fatty acid metabolism, mitochondrial function, and energy regulation pathways. Overall, this study provides a comprehensive profile of transcriptomic and proteomic alterations in pigs and offers a novel theoretical foundation for further investigating the molecular mechanisms behind heterosis-related meat quality traits in Songlei black pigs.

## Data Availability

The datasets presented in this study can be found in online repositories. The raw sequencing data have been deposited in the Genome Sequence Archive (GSA) at the National Genomics Data Center, China National Center for Bioinformation, under accession number CRA035339. The data are publicly accessible via: https://ngdc.cncb.ac.cn/gsa/browse/CRA035339.

## Footnotes

1
https://cnsknowall.com/
