# Supplementary material for: Integrating transcriptome and proteome profiles to compare carcass and meat quality traits between Songliao and Songlei black pigs
Source: Front Nutr. 2026 Jan 29;13:1710841. doi: 10.3389/fnut.2026.1710841 (PMC12903774; doi:10.3389/fnut.2026.1710841)
Supplement: Supplementary file 2 [file Table_2.docx]

| Ingredients | Content |
| --- | --- |
| Corn | 78 |
| Soyean meal | 12 |
| Wheat bran | 6.3 |
| Soybean oil | 0.68 |
| Limestone | 0.91 |
| CaHPO4 | 0.45 |
| NaCl | 0.22 |
| Lys | 0.29 |
| Met | 0.03 |
| Thr | 0.1 |
| Try | 0.02 |
| Premix  Total | 1  100 |
| Calculated nutrition content^1^ |  |
| net energy（MJ/Kg） | 10.35781 |
| CP (SID)^2^ | 10.0625 |
| CF | 2.38 |
| EE | 3.9544 |

S1. Composition and nutrient levels of the basal diet ( %，air-dry basis)

^1^The data are calculated according to NY/T 65-2021standards.

^2^SID = standardized ileal digestible values.
